# Relationship of Depth Adaptation Between Disparity-Specified Plaids and Their Components

**DOI:** 10.1177/2041669518799763

**Published:** 2018-09-13

**Authors:** Shufang He, Hiroaki Shigemasu

**Affiliations:** Graduate School of Engineering, Kochi University of Technology, Japan; School of Information, Kochi University of Technology, Japan

**Keywords:** cyclopean-oriented filter, depth adaptation, disparity-defined plaids, disparity-defined gratings, stereopsis

## Abstract

In the luminance domain, studies show that perceived contrasts of plaids are a nonlinear summation of their components. In the disparity domain, perceived depth has been studied by using a depth adaptation paradigm with simple surfaces; however, the relationship between depth adaptation between plaids and their components has not been investigated. To clarify this, combinations of disparity-defined horizontal corrugation (marked as *horizontal*) and disparity-defined plaids as adaptor-probe pairs were used. Three experiments were performed: The first two compared the aftereffects between horizontal-horizontal and plaid-horizontal pairs (Comparison 1) and between horizontal-plaid and plaid-plaid pairs (Comparison 2). Experiments 1 and 2 controlled the plaids to have the same and doubled peak-to-trough amplitudes as the horizontal corrugation, respectively. In Comparison 1, the horizontal or horizontally oriented component of the plaids was adapted. In Comparison 2, the plaid adaptor or horizontally oriented component of the plaid test stimuli was adapted. Thus, depth adaptation may be linked to cyclopean-oriented depth-from-disparity bandpass filters. The depth adaptation degree was determined by the adaptation of amplitudes of the similar oriented channels between the adaptation and test stimuli. Experiment 3 compared the aftereffects between noise-horizontal and horizontal-horizontal pairs. Since the noise adaptor contained multispatial frequency channels, only the channels with similar spatial frequencies as the horizontal corrugation were adapted, thus causing smaller depth aftereffects.

## Introduction

An adaptation effect refers to the change of perception after exposure to a specific stimulus and can be used to explore underlying mechanisms involved in visual processes (Carandini, 2000). The adaptation paradigm has been used in numerous studies of depth perception for decades (Ryan & Gillam, 1993; Schumer & Ganz, 1979). Domini, Adams, and Banks (2001) manipulated the viewing distances of adaptation and test stimuli to investigate whether depth adaptation to curved surfaces is a second-order disparity-specified or percept-specified shape-level process. Based on the principle that perceived curvature from certain disparity stimuli is distance dependent, these researchers examined depth aftereffects using four combinations of 20 - and 80-cm viewing distances as adaptor-probe pairs. If the process is disparity specified, a change in the viewing distance between the adaptor and test stimuli will not cause a significant difference in the aftereffects; if the process is a shape-level process, there will be a significant difference. Their experimental results showed that there were significant differences in aftereffects between pairs that had different adaptor or probe viewing distances, suggesting the distance-dependent process and indicating that shape-level adaptation was involved. Similarly, Berends, Liu, and Schor (2005) examined the mechanism of stereo-slant perception based on the principle that percept-specified slant adaptation is viewing distance dependent. These researchers fixed the adaptation stimulus at a distance of 57 cm and changed the position of the test stimulus to various distances. Results showed that different aftereffects were induced with different viewing distances, suggesting that percept-specified adaptation occurred. Both studies therefore demonstrated the shape-level depth adaptation process. In regard to disparity-specified adaptation, Berends and Erkelens (2001) manipulated the adapting stimuli to be perceived as a fronto-parallel plane by changing the vertical disparity while fixing the horizontal disparities and used test stimuli with only horizontal disparity. Although the perceived adaptation stimuli were fronto-parallel, the test stimuli to be perceived as fronto-parallel were significantly different from zero horizontal disparity. These researchers concluded that the disparity per se, not the perceived depth, was adapted by the visual system. Yan and Shigemasu (2015) dynamically changed the location, size, and depth of spherical adaptation stimuli and found that both disparity- and percept-specified processes were involved in stereo-curvature adaptation.

Although these studies investigated the disparity or shape-level depth adaption with simple stimuli, the relationship of depth adaptation between objects and their components has not been investigated. In the luminance domain, Georgeson and Shackleton (1994) investigated the thresholds of perceived contrast of luminance-defined gratings and luminance-defined plaids and found the perceived contrast of plaids was a nonlinear summation of their different-oriented components. In the disparity domain, Hibbard and Langley (1998) used disparity-defined plaids, and their component sinusoidal gratings to detect the slant and inclination thresholds, and found that the thresholds of plaids could be predicted by the thresholds of their components. Moreover, Bowd, Donnelly, Shorter, and Patterson (2000) used two moving square-wave gratings to produce plaid test stimuli and compared the adaptation coherence ratio by adapting to plaids and gratings within or across luminance and disparity domains. Results showed that there was a cross-domain coherence adaptation, which meant using moving disparity-defined plaids or their components as adaptor and luminance-defined plaids as test stimuli, could cause a certain amount of coherence aftereffects. This provided evidence for a common underlying mechanism of perceived motion in luminance and disparity domains.

Tyler (1975) demonstrated hierarchical processes of neural adaptation, which involved the retinal level luminance adaptation, contrast adaptation, cyclopean depth adaptation, and hypercyclopean adaptation. In his study, he used sinusoidal corrugations as stimuli to investigate the adaptation, but he mainly focused on the disparity-defined hypercyclopean adaptation such as tilt or size-related adaptation or the cyclopean depth adaptation of sinusoidal corrugation per se. The relationship of depth adaptation between plaids and their components has not been investigated.

In this study, we aim to investigate the relationship between plaids and their component gratings within the amounts of depth adaptation. Three experiments were conducted: For Experiments 1 and 2, we used combination of disparity-defined horizontal corrugation (marked as *horizontal*) and disparity-defined plaids as adaptor-probe pairs, and compared the aftereffects between the horizontal-horizontal pair and the plaid-horizontal pair and the aftereffects between the horizontal-plaid pair and plaid-plaid pair. The disparity-defined plaids are a combination of two orthogonal gratings (horizontally and vertically oriented); thus, the peak-to-trough amplitude was doubled after combination. In Experiment 1, we controlled the plaids that had the same peak-to-trough amplitude as the horizontally oriented gratings; thus, each component had only half the peak-to-trough amplitude. In Experiment 2, the plaids had double the peak-to-trough amplitude as the horizontally oriented grating. This meant that each component of plaids had the same peak-to-trough amplitude as the horizontally oriented gratings. We quantitatively changed the peak-to-trough amplitude of the plaids and investigated how the amount of depth aftereffects changed. In Experiment 3, we verified whether differences in depth aftereffects were induced by the adaptors with or without certain surfaces. To do so, we used horizontally oriented corrugation and noise-shape as adaptors and manipulated the two adaptors that had the same peak-to-trough amplitudes and the same crossed and uncrossed disparities, but the noise-adaptor had randomly distributed disparity dots without a certain surface. In all experiments, we dynamically changed the phase of the stimuli to prevent local slant and curvature adaptation.

We hypothesize that either the amount of depth adaptation is linked to cyclopean-oriented depth-from-disparity bandpass filters or it is linked to the peak-to-trough amplitude of the pooled stimuli. For the former hypothesis, since the plaids consist of two orthogonal gratings (the horizontally and vertically oriented gratings), the oriented disparity-defined bandpass filters determine that, in the horizontally oriented test stimulus condition, the horizontally oriented adaptor or the horizontally oriented component of the plaid adaptor will be adapted. In the plaid test stimulus condition, the plaid adaptor will be adapted in the plaid-plaid condition, while the horizontally oriented component will be adapted in the horizontal-plaid condition. As a result, in Experiment 1, the half plaid-horizontal pair and half plaid-half plaid pair will cause smaller amounts of aftereffects than the horizontal-horizontal pair and horizontal-half plaid pair, respectively. In Experiment 2, the plaid-horizontal pair and plaid-plaid pair will cause similar amounts of aftereffects as the horizontal-horizontal pair and horizontal-plaid pairs, respectively. In Experiment 3, since the noise-adaptor was random white noise, which contained multiple spatial frequencies and limited amplitude for each spatial frequency channel, during adaptation, only the channels with similar spatial frequency to the horizontally oriented test stimuli will be adapted. Thus, the noise-horizontal pair will cause less adaptation than the horizontal-horizontal pair. However, if the latter hypothesis is true, in Experiment 1, the half plaid-horizontal pair and half plaid-half plaid pair will cause similar amounts of aftereffects as the horizontal-horizontal pair and horizontal-half plaid pair, respectively, because of the same peak-to-trough amplitude in each comparison pair. In Experiment 2, the plaid-horizontal pair and plaid-plaid pair will cause larger amounts of aftereffects than the horizontal-horizontal pair and horizontal-plaid pair, respectively, because of the larger amounts of peak-to-trough amplitude in the plaid adaptor.

## Experiment 1

### Methods

#### Participants

Ten students aged 20 to 35 years (5 men, mean age: 21.7) from Kochi University of Technology were recruited as participants. All had normal or corrected-to-normal vision. Nine of them passed the stereo perception and stereo acuity test (less than 1 arcmin) with our own program; one male participant had a relatively low score on the stereo acuity test (< 50% correct rate) and was excluded from the data analysis. All participants were naïve to the purpose of the experiment. All experiments and procedures were approved by the Research Ethics Committee of Kochi University of Technology and conformed to the tenets of the Declaration of Helsinki. Written informed consent was obtained from all participants prior to experiments.

#### Apparatus

Visual stimuli were presented on a 22-in. CRT color display (RDF223H; Mitsubishi, Tokyo, Japan) with 1024 × 768 resolution and 120 Hz frame refresh rate. The luminance of the display was measured using a CS-100 A colorimeter (Minolta, Japan) and linearized using look-up table method. We created a program using Matlab (Mathworks, Natick, MA, USA) with PsychToolbox Version 3 to present the experimental stimuli (Brainard, 1997; Pelli, 1997). During the experiments, participants sat in a dark room fronto-parallel to the surface of the display and observed the stimuli via a pair of stereoscopic wireless LCD glasses (NuVision 60GX; MacNaughton, Inc., OR, USA). The refresh rate of the LCD glasses was 120 Hz, so the frame rate was 60 Hz for each eye. No flicker was reported. A chin rest was used to prevent head movement.

#### Stimuli

Random dot stereograms with horizontal disparity were used for the stimuli. Anti-aliased pseudo-random white dots (29.7 cd/m^2^) were presented on a gray background (9.9 cd/m^2^). Because of the low contrast and gray background of the stimuli, the cross-talk effect was negligible. The dot patterns of the adaptation stimuli were randomly changed every 200 ms, and the density of the dot pattern was 30.6 dots/deg^2^.

At the center of the display, a nonius fixation with lower part T- and upper part reversed T-shape was shown to the left and right eyes separately. To ensure eye vergence, participants were asked to maintain the vertical lines of the two T parts collinearly, and the horizontal lines overlapped during the entire experimental procedure. With the correct vergence, the nonius was perceived as a cross. The lengths of both the horizontal and vertical lines were 1.17 arcdeg.

#### Procedure

**Figure 1.**
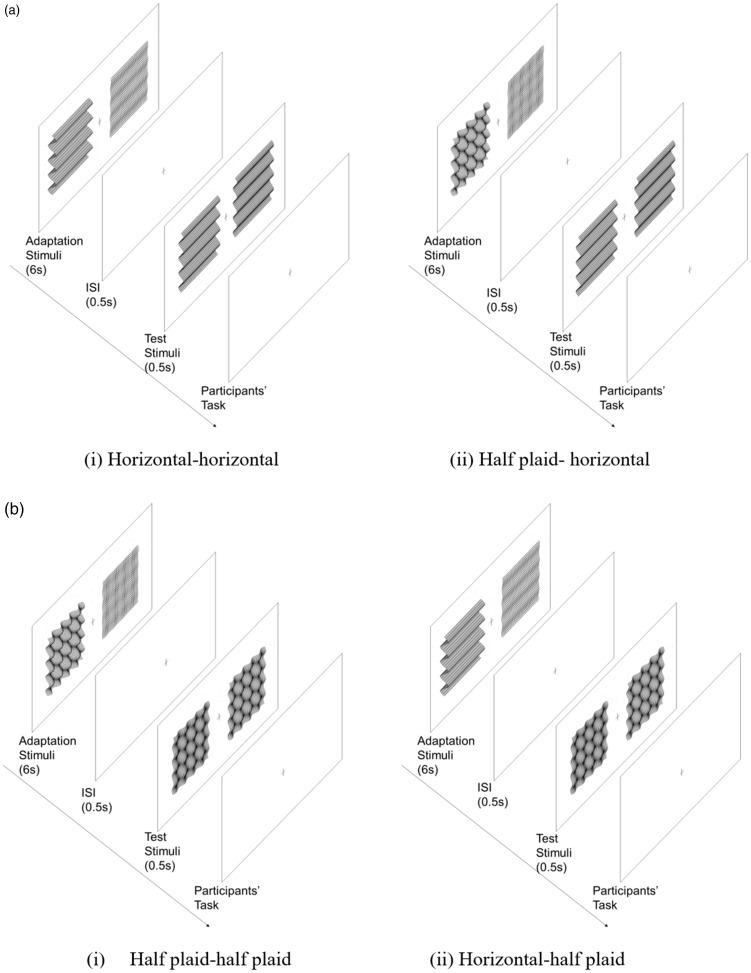
A schematic representation of the test procedure in Experiment 1. (a) The horizontally oriented test stimulus condition and (b) half plaid test stimulus condition. In all conditions, both adaptors were presented for 6 s. After a 0.5-s time interval during which a blank gray background was displayed, the test stimulus was presented on one side, and a comparison stimulus was presented on the other side for 0.5 s simultaneously. The comparison stimulus had a fixed amplitude (12.1 arcmin), whereas the test stimulus had nine levels of amplitudes (9.1–15.2 arcmin with constant intervals) and were presented in a random order. No one could predict what would be presented in the next trail. The positions of the test and comparison stimuli were presented on the left and right sides of the display in a counterbalanced random order. Each participant’s task was to judge which side had the larger amplitude and to report their choice by button press based on two-alternative forced-choice methods. No feedback on correctness was given. After participants made their choice, the next trial was triggered automatically. ISI = Inter Stimulus Interval.

In Experiment 1, we aimed to investigate the relationship of depth adaptation between disparity-defined plaid pattern and their components. Combinations of horizontally oriented corrugation and plaids were used as adaptor-probe pairs and the changes in depth between horizontal-horizontal and plaid-horizontal pairs were compared as the horizontally oriented test stimulus condition (Figure 1(a)), and the changes in depth between plaid-plaid and horizontal-plaid pairs as the plaid test stimulus condition (Figure 1(b)). The plaids are the combination of two orthogonal gratings; thus, the amplitude (of the disparity between peak and trough) will be doubled when the two orthogonal components are linearly added up. To control for plaids with the same peak-to-trough amplitude as the horizontal corrugation, we defined the amplitudes of the horizontally and vertically oriented components as half the amplitude of the horizontal corrugation. To differentiate the plaids used here with those used in Experiment 2, we used the term *half plaid* in Experiment 1 to represent the plaid stimuli.

Therefore, the horizontally oriented grating and the half plaid are shown in Equations (1) and (2), respectively:

(1) $${\text{S}}_{\text{Hor}}=\text{A}\times \text{sin}(2\pi y+\phi )$$

(2) $${\text{S}}_{\text{halfplaid}}=0.5\times \text{A}\times (\text{sin}(2\pi y+\phi )+\text{sin}(2\pi x+\phi ))$$

where A is the amplitude with the value shown in Table 1 at different stimulus conditions; *x* and *y* are the horizontal and vertical positions on the CRT display, respectively, and the spatial frequencies of horizontal corrugation and plaid components were 0.25 cpd; ϕ is the phase, which randomly changes each 200 ms. The size of the stimuli on each side was 14.0 × 14.0 arcdeg.

**Table 1.** Parameters of the Horizontal Corrugation and Half Plaid Stimuli.

Stimulus type	Depth amplitude of horizontal corrugation and half plaid stimuli
Adaptation stimulus	
Large	20.2 arcmin
Medium	12.1 arcmin
Small	4.1 arcmin
Test stimulus	9.1–15.2 arcmin (nine levels)
Comparison stimulus	12.1 arcmin

For the left and right eye inputs to produce each kind of stimulus, we shifted a certain amount of disparity to produce a stereo image after binocular fusion. For example, for the horizontally oriented stimulus, the left and right eye inputs were shown as Equations (3) and (4), respectively.

(3) $${\text{S}}_{\text{L}}={\text{S}}_{\text{Hor}}+\Delta \text{d}/2$$

(4) $${\text{S}}_{\text{R}}={\text{S}}_{\text{Hor}}-\Delta \text{d}/2$$

where Δd represents the disparity.

The parameters of the horizontal corrugation and half plaid stimuli are shown in Table 1.

In each adaptor-probe pair, there were large-small and medium-medium adapting amplitude conditions. In each condition, both adaptors were presented for 6 s. After a 0.5-s time interval that included exposure to a blank gray background, the test stimulus was presented on one side, and the comparison stimulus was presented on the other side for 0.5 s simultaneously. The comparison stimulus had a fixed amplitude (12.1 arcmin), whereas the test stimulus had nine levels of amplitudes (9.1–15.2 arcmin with constant intervals) and were presented in a random order. No one could predict what would be presented for next trial. The positions of test and comparison stimuli were presented on the left and right sides of the display in a counterbalanced, random order. A participant’s task was to judge which side had larger amplitude and to report their choices by pressing a button based on a two-alternative forced-choice method. No feedback of correctness was given. After participants made their choice, the next trial was triggered automatically.

In the large-small adaptor condition, after adaptation, the side with the large-amplitude adaptor should cause the amplitude of the probe to appear smaller than the actual value, whereas the side with a small-amplitude adaptor should cause the amplitude of the probe to appear larger. Thus, there will be a perceptual shift. In contrast, in the medium-sized amplitude adaptor condition, the two adaptors have the same amplitudes, which cause the same adaptation effects of the test stimuli and therefore will not induce a perceptual shift.

Before the experiment, participants were trained by using our own practice program. In practice trials, the adaptation step was eliminated to avoid any potential influence on the experimental results, and only the test stimuli were shown. Feedback on correctness was given to participants to give them insight on their own perception. There were 36 practice trials for each stimulus type.

During the experiment, stimuli were presented with two combinations of adaptor-probe pairs in each test stimulus condition (i.e., horizontal-horizontal and half plaid-horizontal pairs as the horizontal test stimulus condition, and half plaid-half plaid and horizontal-half plaid pairs as the half plaid test stimulus condition) and three adaptation-amplitude types (large-, medium-sized, and small-amplitude adaptation conditions). For each condition, there were 216 trials to produce 8 repeats at each test point. Different conditions were block designed and divided into six sessions that were implemented on different days. In each session, blocks were presented subsequently with a 2-min break between each block. All the blocks were counterbalanced within and between subjects.

### Results

**Figure 2.**
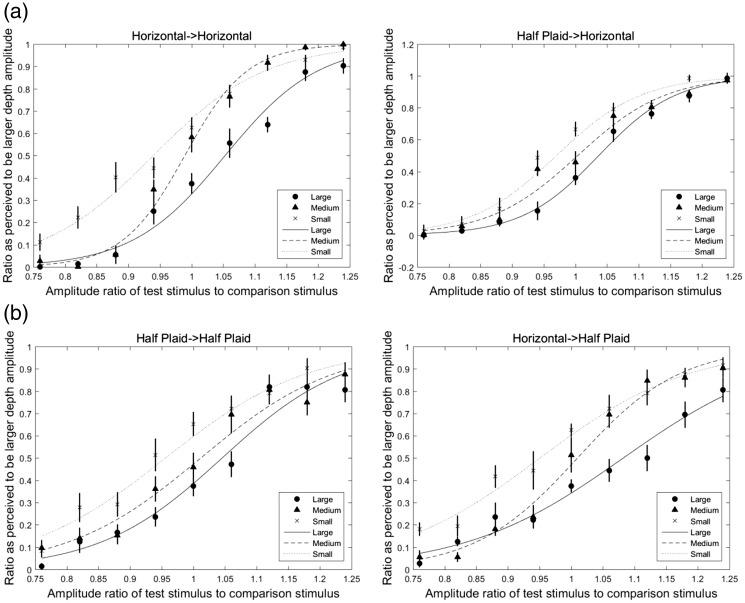
Fitted psychometric sigmoidal curves in horizontally oriented and half plaid test stimulus conditions. (a) The horizontally oriented test stimulus condition, in which the left and right panels represent the horizontal-horizontal and half plaid-horizontal adaptor-probe conditions, respectively and (b) the half plaid test stimulus condition, in which the left and right panels represent the half plaid-half plaid and horizontal-half plaid adaptor-probe conditions, respectively. In all figures, the horizontal axis represents the amplitude ratio of test stimulus to comparison stimulus, and the vertical axis represents the ratio perceived to be the larger depth amplitude after separating the trials that presented test stimuli after the large adaptor from trials that presented stimuli after the small adaptor. The average values of the ratio perceived to be larger depth amplitude after adapting to large-, medium-sized, and small-amplitude adaptors are shown by circle, triangle, and cross symbols, respectively. The fitted curves after adapting to large-, medium-, and small-amplitude adaptors are shown by solid line, dash line, and dot line, respectively. The error bars are the standard errors of the mean by nine participants’ data.

Stimuli were presented with four combinations of adaptor-probe pairs and three adapting-amplitude types. Figure 2 shows the sigmoidal curves as a psychometric function fitted with the average data of nine participants by using the generalized linear fitting method (Kingdom & Prins, 2010) and the Matlab program (Mathworks, Natick, MA, USA).

In the horizontally oriented test stimulus condition as shown in Figure 2(a), the left and right panels show the horizontal-horizontal and half plaid-horizontal adaptor-probe conditions, respectively, and in the half plaid test stimulus condition as shown in Figure 2(b), the left and right panels show the half plaid-half plaid and horizontal-half plaid adaptor-probe conditions, respectively. In all figures, the horizontal axis represents the normalized amplitude of test stimulus, which is calculated by $\frac{\mathrm{Depth}\mathrm{of}\mathrm{test}\mathrm{stimulus}}{\mathrm{Depth}\mathrm{of}\mathrm{comparison}\mathrm{stimulus}}$ with the depth parameters shown in Table 1. The vertical axis represents the ratio perceived to be the larger depth amplitude. Since the test and comparison stimuli were presented randomly after the large or small adaptors in each trial, we separated those trials with test stimuli after the large adaptor from those after the small adaptor; then for each amplitude level of test stimuli (nine levels in total), we calculated the ratio perceived to be a larger depth amplitude than the comparison stimulus. After this calculation, we obtained the results for the three adapting conditions separately. Each test stimulus point was repeated eight times for each participant. The average values of the ratio perceived to be larger depth amplitude after adapting to large-, medium-sized, and small-amplitude adaptors are shown by circle, triangle, and cross symbols, respectively. The fitted curves are shown by solid line, dash line, and dot line, respectively. The error bars are the standard errors of the mean by 10 participants’ data. The fitted curves showed that there were shifts among the large-, medium-sized, and small-amplitude adapting conditions in each adaptor-probe pair.

**Figure 3.**
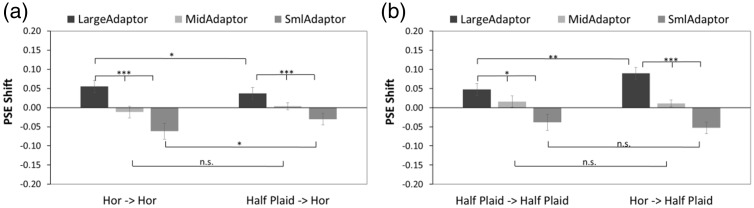
The PSE shift in Experiment 1. (a) Horizontally oriented test stimulus condition. (b) Half plaid test stimulus condition. Significant differences in amplitude-adapting (large-, medium-sized, and small-amplitude adapting conditions) are found in both adaptation conditions. The standard error bars are shown in all conditions. PSE = point of subjective equality.

The 50% point on the fitted psychometric function, namely, point of subjective equality (PSE), was obtained for each participant by calculating the horizontal-axis value (as *amplitude ratio of test stimulus to comparison stimulus*) which corresponded to 0.5 of the vertical-axis value (this was also the *ratio perceived to be the larger depth amplitude*) in each condition as shown in Figure 2 and then subtracting 1 to get the PSE shift accordingly. The results of the PSE shift in each condition are presented in Figure 3.

A 2 (adaptor-probe conditions) × 3 (large, medium-sized, and small adaptation conditions) repeated measure analysis of variance (ANOVA) was used to analyze the PSE shift of the horizontal and plaid test stimulus conditions separately.

In the horizontal test stimulus condition (Figure 3(a)), ANOVA revealed a significant main effect on amplitude type, *F*(2, 16) = 19.88, *p* < .001, generalized η^2 ^= 0.62, and a significant interaction between adaptor-probe pair and amplitude type, *F*(2, 16) = 5.06, *p* = .02, generalized η^2 ^= 0.11, while no significant difference was found in the PSE shift of adaptor-probe pairs, *F*(1, 8) = 4.74, *p* = .06, generalized η^2 ^= 0.03. Significant simple main effects of the adaptor-probe pairs were noted in the large-amplitude adapting condition, *F*(1, 8) = 5.48, *p* = .047, generalized η^2 ^= 0.11, and small-amplitude adapting condition, *F*(1, 8) = 6.05, *p* = .04, generalized η^2 ^= 0.17. Thus, the absolute values of PSE shift are significantly larger in horizontally corrugated adaptor than in half plaid adaptor conditions. No significant difference in the medium-sized amplitude adapting condition was found, *F*(1, 8) = 2.86, *p* = .13, generalized η^2 ^= 0.09. Multiple comparison tests showed significant differences between each two amplitude adapting conditions in the horizontal-horizontal condition (*p* < .001 between the large- and small-amplitude adapting conditions, *p* < .001 between the large- and medium-sized amplitude adapting conditions, and *p* = .02 between the medium-sized and small-amplitude adapting conditions) and in the half plaid-horizontal condition (*p* < .001 between the large- and small-amplitude adapting conditions, *p* = .02 between the large- and medium-sized amplitude adapting conditions, and *p* = .04 between the medium-sized and small-amplitude adapting conditions).

In the half plaid test stimulus condition (Figure 3(b)), ANOVA revealed a significant main effect of amplitude type, *F*(2, 16) = 13.56, *p* < .001, generalized η^2 ^= 0.52, and a significant interaction between adaptor-probe pair and amplitude type, *F*(2, 16) = 3.96, *p* = .04, generalized η^2 ^= 0.07, whereas no significant difference in adaptor-probe pairs was noted, *F*(1, 8) = 1.31, *p* = .29, generalized η^2 ^= 0.01. A significant simple main effect of adaptor-probe pairs was found in the large condition, *F*(1, 8) = 14.48, *p* = .01, generalized η^2 ^= 0.19, whereas no significant difference in the medium-, *F*(1, 8) = 0.10, *p* = .75, generalized η^2 ^= 0.003, or small-amplitude adapting conditions, *F*(1, 8) = 0.52, *p* = .49, generalized η^2 ^= 0.02, was noted. Thus, the absolute value of the PSE shift was found to be significantly larger in horizontally corrugated stimuli than in half plaid adaptor stimuli. Multiple comparison tests showed significant differences in the half plaid-half plaid condition (*p* = .02 between the medium-sized and small-amplitude conditions, *p* = .04 between the large- and small-amplitude conditions) and in the horizontal-plaid condition (*p* < .001 between each of the two amplitude-adapting conditions among the large-, medium-sized, and small-amplitude adaptors).

## Experiment 2

In Experiment 1, we compared the aftereffects of depth adaptation between half plaids and horizontal corrugation and found significant differences in depth adaptation between horizontal-horizontal and half plaid-horizontal pairs and also between half plaid-half plaid and horizontal-half plaid pairs. In this experiment, we did not control the peak-to-trough amplitude of the disparity-defined plaids; thus, the components of plaids had the same peak-to-trough amplitude as that of horizontal gratings. The purpose of this experiment was to examine whether the component of the plaid was relevant to the adaptation effect.

### Methods

#### Participants

Ten participants (21–31 years, mean: 25.6 years, 6 men) took part in this experiment. All of them had normal or corrected-to-normal vision and passed the stereo perception and stereo acuity test (less than 1 arcmin) with our own program. Participants were naïve to the purpose of the experiment and were compensated for their time.

#### Apparatus

The apparatus was the same as in Experiment 1.

#### Stimuli

The peak-to-trough amplitude of disparity-defined plaid adaptors was double the half plaids in Experiment 1, as shown in Equation (5), whereas the disparity-defined horizontal gratings were kept the same as those in Experiment 1.

(5) $${\text{S}}_{\text{Plaid}}=\text{A}\times (\text{sin}(2\pi y+\phi )+\text{sin}(2\pi x+\phi ))$$

where A represents the amplitude with the value shown in the right column in Table 2; *x* and *y* are the horizontal and vertical positions on the CRT display, respectively, and the spatial frequencies of horizontal corrugation and plaid components were 0.25 cpd; ϕ is the phase, which randomly changes each 200 ms. The size of the stimuli on each side was 14.0 × 14.0 arcdeg.

**Table 2.** Parameters of Horizontally Oriented Corrugation and Plaid Stimuli.

Stimulus type	Depth amplitude of horizontally oriented stimuli	Depth amplitude of plaid stimuli
Adaptation stimulus		
Large	20.2 arcmin	40.4 arcmin
Medium sized	12.1 arcmin	24.2 arcmin
Small	4.1 arcmin	8.2 arcmin
Test stimulus	9.1–15.2 arcmin (nine levels)	18.2–30.4 arcmin (nine levels)
Comparison stimulus	12.1 arcmin	24.2 arcmin

#### Procedure

A schematic representation of the procedure is the same as in Figure 1 except that all the half plaids were replaced with plaids in Experiment 2. In each condition, there were 216 trials to produce eight repeats of each test stimuli. The time duration and the procedure were the same as in Experiment 1. Each participant’s task was to judge which side had the larger amplitude. The experiment was block designed, and each session was conducted on a different day.

### Results

**Figure 4.**
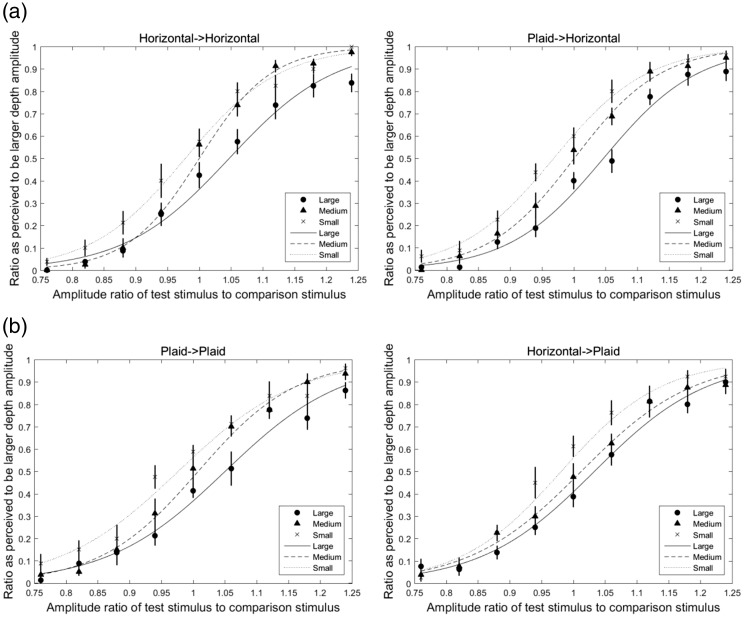
Fitted psychometric sigmoidal curves in horizontal and plaid test stimulus conditions. (a) Horizontally oriented test stimulus condition and (b) plaid test stimulus condition. (a) The horizontally oriented test stimulus condition, in which the left and right panels represent the horizontal-horizontal and plaid-horizontal adaptor-probe conditions, respectively and (b) the plaid test stimulus condition, in which the left and right panels represent the plaid-plaid and horizontal-plaid adaptor-probe conditions, respectively. In all figures, the horizontal axis represents the amplitude ratio of test stimulus to comparison stimulus, and the vertical axis represents the ratio perceived to be the larger depth amplitude after separating the trials that presented test stimuli after the large adaptor from trials that presented stimuli after the small adaptor. The average values of the ratio perceived to be larger depth amplitude after adapting to large-, medium-sized, and small-amplitude adaptors are shown by circle, triangle, and cross symbols, respectively. The fitted curves after adapting to large-, medium-sized, and small-amplitude adaptors are shown by solid line, dash line, and dot line, respectively. The error bars are the standard errors of the mean by 10 participants’ data.

Stimuli were presented with four combinations of adaptor-probe pairs and three adaptation-amplitude types (large-, medium-sized, and small-amplitude adapting conditions). We used the same method as in Experiment 1 to calculate the ratio perceived to be the larger depth amplitude. Figure 4 shows the sigmoidal curves as a psychometric function fitted with the average data of 10 participants.

In the horizontally oriented test stimulus condition as shown in Figure 4(a), the left and right panels show the horizontal-horizontal and plaid-horizontal adaptor-probe conditions, respectively, and in the plaid test stimulus condition as shown in Figure 4(b), the left and right panels show the plaid-plaid and horizontal-plaid adaptor-probe conditions, respectively. In all figures, the horizontal axis represents the normalized amplitude of test stimulus, which is calculated by $\frac{\mathrm{Depth}\mathrm{of}\mathrm{test}\mathrm{stimulus}}{\mathrm{Depth}\mathrm{of}\mathrm{comparison}\mathrm{stimulus}}$ with the depth parameters shown in Table 2. The vertical axis represents the ratio perceived to be the larger depth amplitude. We also separated those trials with test stimuli after the large adaptor from those after the small adaptor using the same method as in Experiment 1, and then for each amplitude level of test stimuli (nine levels in total) calculated the ratio perceived to be larger depth amplitude than the comparison stimulus. The average values of the ratio perceived to be larger depth amplitude after adapting to large-, medium-sized, and small-amplitude adaptors are shown by circle, triangle, and cross symbols, respectively. The fitted curves after adapting to large-, medium-sized, and small-amplitude adaptors are shown by solid line, dash line, and dot line, respectively. The fitted curves showed that there were shifts among the large-, medium-sized, and small-amplitude adapting conditions in each adaptor-probe pair. The error bars are the standard errors of the mean by 10 participants’ data.

**Figure 5.**
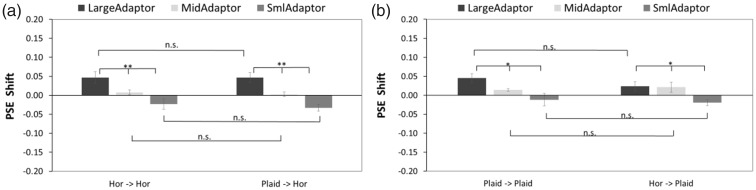
The PSE shift in Experiment 2. (a) Horizontally oriented test stimulus condition. (b) Plaid test stimulus condition. Significant differences in amplitude-adapting (large, medium-sized, and small-amplitude adapting conditions) are found in both conditions. The standard error bars are shown in all conditions. PSE = point of subjective equality.

The PSE shift was calculated in the same method as described in the Results section of Experiment 1 and presented in Figure 5. A 2 (adaptor-probe conditions) × 3(large-, medium-, and small-adaptation conditions) repeated measure ANOVA was used to analyze the PSE shifts of the horizontal and plaid test stimulus conditions separately.

In the horizontal test stimulus condition (Figure 5(a)), the ANOVA revealed neither a significant main effect of adaptor-probe pairs, *F*(1, 9) = 2.16, *p* = .18, generalized η^2 ^= 0.01, nor a significant interaction between adaptor-probe pairs and amplitude types, *F*(2, 18) =0.19, *p* = .82, generalized η^2 ^= .003. However, a significant difference among adaptation amplitude types was found, *F*(2, 16) = 10.02, *p* = .001, generalized η^2 ^= 0.45.

In the plaid test stimulus condition (Figure 5(b)), ANOVA revealed neither a significant main effect of adaptor-probe pairs, *F*(1, 9) = 3.90, *p* = .08, generalized η^2 ^= 0.01, nor a significant interaction between adaptor-probe pairs and amplitude types, *F*(2, 18) = 1.34, *p* = .29, generalized η^2 ^= 0.03. However, a significant difference among adaptation amplitude types was found, *F*(2, 18) = 4.85, *p* = .02, generalized η^2 ^= 0.25.

## Experiment 3

In Experiments 1 and 2, we respectively manipulated the peak-to-trough amplitudes of the half plaids and plaids to investigate how the depth aftereffects changed. In Experiment 3, we aimed to verify whether changes in depth aftereffects were induced between the adaptors with and without certain surfaces. To do so, we used horizontal corrugation and noise-shape as adaptors.

### Methods

#### Participants

The 10 participants of Experiment 1 took part in Experiment 3. We also excluded the data of one participant because of their low stereo acuity test score as explained in Experiment 1.

#### Apparatus

The apparatus was the same as in Experiments 1 and 2.

#### Stimuli

**Figure 6.**
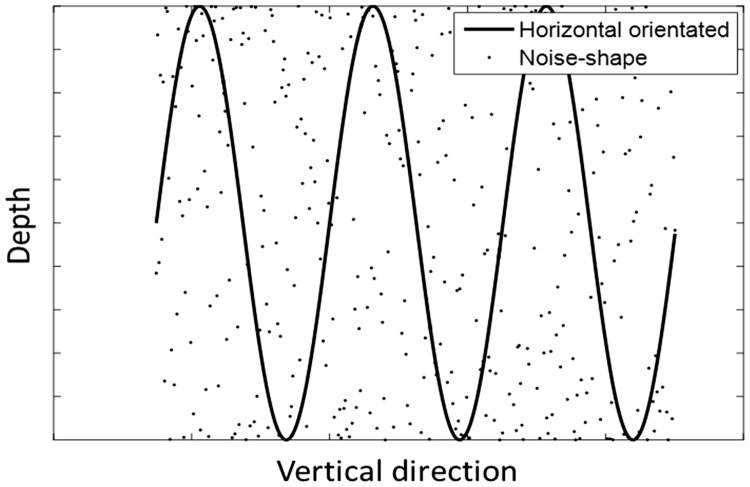
A schematic representation of the noise and horizontally oriented adaptors. The noise and horizontally oriented adaptors are shown in dots and a solid line, respectively.

The noise-shape adaptor had the same peak-to-trough amplitudes and the same crossed and uncrossed disparities as the horizontally oriented corrugation adaptor, but the disparity distribution of the noise adaptor was in random positions without a continuous surface. The horizontal corrugation had the same parameters as presented in Tables 1 and 2. The schematic representation of the stimuli is shown in Figure 6.

#### Procedure

**Figure 7.**
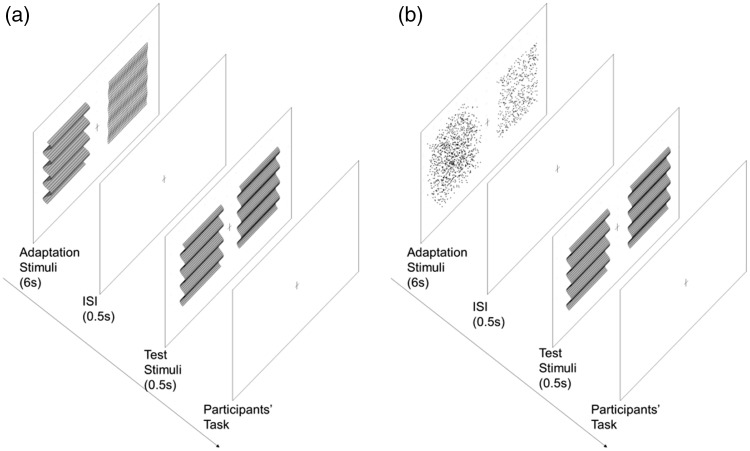
A schematic representation of the procedure for Experiment 2. (a) Horizontal-adapting condition and (b) noise-adapting condition. The experimental procedure was the same as explained in Experiment 1 in Figure 1. ISI = Inter Stimulus Interval.

A schematic representation of the procedure shown in Figure 7 is the same as that explained in Figure 1, thus was omitted here. In each condition, there were 216 trials to produce eight repeats at each test stimuli. The time duration and the procedure were the same as in Experiment 1. Each participant’s task was to judge which side had the larger amplitude. The experiment was block designed, and each session was conducted on a different day.

### Results

In Experiment 3, the stimuli were presented with two combinations of adaptor-probe pairs (horizontal-horizontal and noise-horizontal) and three adaptation-amplitude types (large-, medium-sized, and small-amplitude adapting conditions).

**Figure 8.**
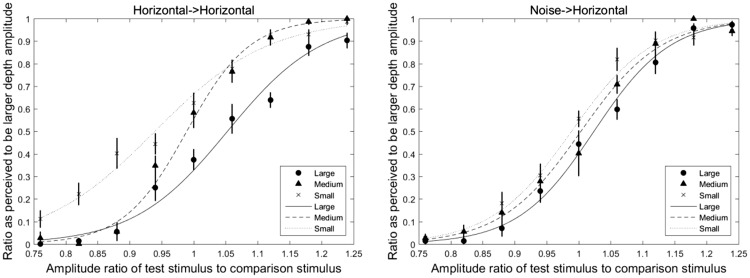
Fitted psychometric sigmoidal curves in horizontally oriented and noise adaptor conditions. The left and right panels represent the horizontal-horizontal and noise-horizontal adaptor-probe conditions, respectively. In all figures, the horizontal axis represents the amplitude ratio of test stimulus to comparison stimulus, and the vertical axis represents the ratio perceived to be the larger depth amplitude after separating the trials that presented test stimuli after the large adaptor from trials that presented stimuli after the small adaptor. The average values of the ratio perceived to be larger depth amplitude after adapting to large-, medium-sized, and small-amplitude adaptors are shown by circle, triangle, and cross symbols, respectively. The fitted curves after adapting to large-, medium-sized, and small-amplitude adaptors are shown by solid line, dash line, and dot line, respectively. The error bars are the standard errors of the mean by 10 participants’ data.

The psychometric sigmoidal curves were fitted, and the PSE shift was calculated from data of nine participants. Figure 8 shows the fitted psychometric sigmoidal curves from the average data of participants. The left and right panels show the horizontal-horizontal and plaid-horizontal adaptor-probe conditions, respectively. In both figures, the horizontal axis represents the normalized amplitude of test stimulus, and the vertical axis represents the ratio perceived to be the larger depth amplitude calculated with the same method as in Experiments 1 and 2. The average values of the ratio perceived to be larger depth amplitude after adapting to large-, medium-sized, and small-amplitude adaptors are shown by circle, triangle, and cross symbols, respectively. The fitted curves after adapting to large-, medium-, and small-amplitude adaptors are shown by solid line, dash line, and dot line, respectively. The fitted curves showed that there were shifts among the large, medium-sized, and small-amplitude adapting conditions in each adaptor-probe pair. The error bars are the standard errors of the mean by nine participants’ data.

**Figure 9.**
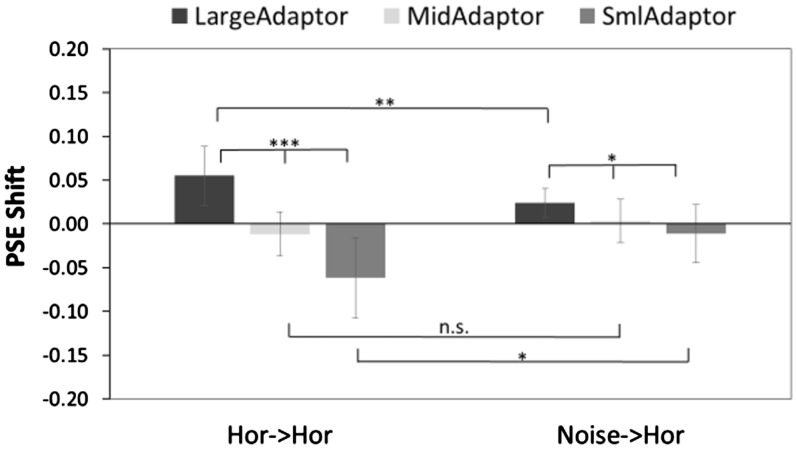
The PSE shift in Experiment 3. Significant differences in adaptation amplitude (large, medium-sized, and small-amplitude adapting conditions) were found in both adaptation conditions. Significant differences of depth adaptation were also noted between the two adaptation conditions in both large- and small-amplitude adapting conditions but not in medium-sized amplitude adapting condition. PSE = point of subjective equality.

A 2 (adaptor-probe conditions) × 3(large-, medium-, and small-adaptation conditions) repeated measure ANOVA was used to analyze the PSE shift of the horizontal and noise adaptation conditions. The ANOVA revealed a significant main effect of adaptor-probe pairs, *F*(1, 8) = 5.37, *p* = .05, generalized η^2 ^= 0.05, amplitude type, *F*(2, 16) = 15.62, *p* < .001, generalized η^2 ^= 0.53, and a significant interaction between the adaptor conditions and adaptor-probe pairs, *F*(2, 16) = 9.26, *p* < .001, generalized η^2 ^= 0.25. Significant simple main effects of adaptation amplitude type were observed in both adaptation conditions (horizontal adaptor: *F*(2, 16) = 17.60, *p* < .001, generalized η^2 ^= 0.68; noise adaptor: *F*(2, 16) = 3.81, *p* = .04, generalized η^2 ^= 0.26). Significant simple main effects of depth adaptation were found with large-amplitude adapting condition, *F*(1, 8) = 11.44, *p* = .01, generalized η^2 ^= 0.28, and small-amplitude adapting condition, *F*(1, 8) = 9.55, *p* = .01, generalized η^2 ^= 0.32, whereas no significant difference was observed with medium-sized amplitude adapting condition, *F*(1, 8) = 3.34, *p* = .10, generalized η^2 ^= 0.14 (Figure 9).

## General Discussion

We conducted three experiments to investigate the relationship of depth adaptation between plaids and their component gratings. In Experiments 1 and 2, we manipulated different peak-to-trough amplitudes of adaptors and test stimuli to investigate whether the peak-to-trough amplitude or the component of the plaid was relevant to the adaptation effect. In Experiment 1, we examined the depth aftereffects between horizontal-horizontal and half plaid-horizontal pairs and also between half plaid-half plaid and horizontal-half plaid pairs. Results showed significant differences in the depth aftereffects of each comparison. In Experiment 2, we investigated the depth aftereffects between horizontal-horizontal and plaid-horizontal pairs and also between plaid-plaid and horizontal-plaid pairs. Results showed no significant differences in the depth aftereffects of each comparison.

In regard to the disparity and shape-level depth adaptation, previous studies (Berends et al., 2005; Domini et al., 2001) used viewing distance dependency to distinguish whether the adaptation was a shape-level process. In our study, we did not manipulate the different viewing distances of the adaptation and test stimuli but changed the peak-to-trough amplitudes of the plaid stimuli. This might cause a change in perception in both disparity and shape levels, since the average crossed or uncrossed disparities, and also the perceived shapes (curvatures) of the plaids, were changed at different plaid amplitudes, thus causing different changes in depth aftereffects. In addition, as the gratings were components of plaids, it was difficult to differentiate the gratings (or components of the plaids) based on shape or to demonstrate the disparity or shape-level depth adaptation; we could not identify what percent was caused by disparity versus the percent caused by the shape-level process.

However, from a cyclopean depth adaptation view, these results might indicate that depth adaptation was linked to cyclopean-oriented depth-from-disparity bandpass filters, as our first hypothesis predicts, and the amount of depth adaptation is determined by the relevance of amplitudes between the adaptation and test stimuli in each channel. In the horizontally oriented test stimulus condition, the horizontal adaptor or the horizontally oriented components of the plaid adaptor were adapted to the horizontal test stimuli. In Experiment 1, the horizontally oriented components of half plaids had half the peak-to-trough amplitude (20.2/2 = 10.1 arcmin in the large-amplitude adaptor and 4.1/2 = 2.05 arcmin in the small-amplitude adaptor conditions) of the horizontally corrugated adaptor. The 10.1 to 2.05 arcmin in the large-small adaptation pair (half plaid-horizontal adaptor-probe condition) caused less aftereffects than the 20.2 to 4.1 arcmin in the large-small adaptation pair (horizontal-horizontal adaptor-probe condition) when using 12.1 arcmin as the amplitude of the test stimuli. However, in Experiment 2, the horizontally oriented components of plaids had the same peak-to-trough amplitude (20.2 arcmin with the large-amplitude adaptor and 4.1 arcmin with the small-amplitude adaptor) as that of the horizontally oriented adaptor and thus caused a similar amount of depth adaptation. Similarly, in the half plaid-half plaid test stimulus condition in Experiment 1, the adaptor and probe had the same shape and same components (20.2 arcmin with the large-amplitude adaptor and 4.1 arcmin with the small-amplitude adaptor), and thus the amount of depth adaptation was similar to the horizontal-horizontal pair. However, for the horizontal-half plaid pair in Experiment 1, the horizontally oriented components in half plaids had half the peak-to-trough amplitude (10.1 arcmin with the large-amplitude adaptor and 2.05 arcmin with the small-amplitude adaptor). This may have caused a smaller change in depth aftereffect when comparing using the horizontally oriented adaptor. However, in the horizontal-plaid pair in Experiment 2, the horizontally oriented component of the plaid adaptor had the same peak-to-trough amplitude as the horizontally oriented adaptor and thus caused a similar amount of depth aftereffects. Meanwhile, based on earlier results, we also disproved the second hypothesis, namely, that the amount of depth adaptation was linked to the peak-to-trough amplitude of the pooled stimuli.

In Experiment 3, we compared the aftereffects between noise-horizontal and horizontal-horizontal pairs, and the results showed a significant difference in depth adaptation between these two pairs. The noise-adaptor was random white noise, which contained multiple spatial frequencies and limited amplitude for each spatial frequency channel. During adaptation, only the channels with similar spatial frequency to the horizontally oriented test stimuli were adapted and thus caused a much smaller depth adaptation when compared with the horizontally oriented adaptor.

Previous studies (Tyler, 1975; Tyler & Kontsevich, 1995) demonstrated the hierarchical processes of neural adaptation and used sinusoidal corrugations to examine the cyclopean depth aftereffect. However, these studies did not investigate the relationship of depth adaptation between plaids and gratings. In this study, we compared the cyclopean depth aftereffects between different peak-to-trough amplitudes of plaids and gratings and found that depth adaptation was linked to cyclopean-oriented depth-from-disparity bandpass filters. Furthermore, the amount of depth adaptation was determined by the relevance of amplitudes between the adaptation and test stimuli in each channel.
